# Linkage Disequilibrium and Effective Population Size of Buffalo Populations of Iran, Turkey, Pakistan, and Egypt Using a Medium Density SNP Array

**DOI:** 10.3389/fgene.2021.608186

**Published:** 2021-12-07

**Authors:** Shirin Rahimmadar, Mokhtar Ghaffari, Mahdi Mokhber, John L. Williams

**Affiliations:** ^1^ Department of Animal Science, Faculty of Agricultural Science, Urmia University, Urmia, Iran; ^2^ Davies Research Centre, School of Animal and Veterinary Sciences, University of Adelaide, Roseworthy, SA, Australia; ^3^ Department of Animal Science, Food and Nutrition, Università Cattolica Del Sacro Cuore, Piacenza, Italy

**Keywords:** water buffalo, linkage disequilibrium, LD phase persistency, NE, linkage disequilibrium, LD phase persistency

## Abstract

Linkage disequilibrium (LD) across the genome provides information to identify the genes and variations related to quantitative traits in genome-wide association studies (GWAS) and for the implementation of genomic selection (GS). LD can also be used to evaluate genetic diversity and population structure and reveal genomic regions affected by selection. LD structure and Ne were assessed in a set of 83 water buffaloes, comprising Azeri (AZI), Khuzestani (KHU), and Mazandarani (MAZ) breeds from Iran, Kundi (KUN) and Nili-Ravi (NIL) from Pakistan, Anatolian (ANA) buffalo from Turkey, and buffalo from Egypt (EGY). The values of corrected *r* ^2^ (defined as the correlation between two loci) of adjacent SNPs for three pooled Iranian breeds (IRI), ANA, EGY, and two pooled Pakistani breeds (PAK) populations were 0.24, 0.28, 0.27, and 0.22, respectively. The corrected *r* ^2^ between SNPs decreased with increasing physical distance from 100 Kb to 1 Mb. The LD values for IRI, ANA, EGY, and PAK populations were 0.16, 0.23, 0.24, and 0.21 for less than 100Kb, respectively, which reduced rapidly to 0.018, 0.042, 0.059, and 0.024, for a distance of 1 Mb. In all the populations, the decay rate was low for distances greater than 2Mb, up to the longest studied distance (15 Mb). The *r* ^2^ values for adjacent SNPs in unrelated samples indicated that the Affymetrix Axiom 90 K SNP genomic array was suitable for GWAS and GS in these populations. The persistency of LD phase (PLDP) between populations was assessed, and results showed that PLPD values between the populations were more than 0.9 for distances of less than 100 Kb. The Ne in the recent generations has declined to the extent that breeding plans are urgently required to ensure that these buffalo populations are not at risk of being lost. We found that results are affected by sample size, which could be partially corrected for; however, additional data should be obtained to be confident of the results.

## Introduction

Recognizing and protecting the genetic diversity of domestic species is important in the development of breeding strategies (Al-Mamun et al., 2015; Wultsch et al., 2016). Recent progress in the field of genome sequencing has increased the availability of genomic data, which has facilitated the assessment of the genetic diversity and population structure (Vonholdt et al., 2010; Decker et al., 2014) using parameters such as population admixture, linkage disequilibrium (LD), and effective population size (Ne) (Al-Mamun et al., 2015).

The non-random association between alleles at different loci is referred to as LD or gametic phase disequilibrium. Knowledge of the pattern of LD in a population is an important prerequisite for GWAS, exploring population structures, and implementing genomic selection (GS) (Niu et al., 2016). The pattern of LD can be used to estimate the rate of genetic drift, level of inbreeding, and the effects of evolutionary forces such as mutation, selection, and migration (Shin et al., 2013). There have been studies of LD in several livestock species, including cattle (McKay et al., 2007; Karimi et al., 2015; Biegelmeyer et al., 2016; Jemaa et al., 2019), buffalo (Mokhber et al., 2019a; Deng et al., 2019; Lu et al., 2020), pig (Badke et al., 2012; Wang et al., 2013), sheep (Meadows et al., 2008), goat (Brito et al., 2015), chicken (Qanbari et al., 2010; Fu et al., 2015), horse (Corbin et al., 2010), dog (Pfahler & Distl., 2015), and cat (Alhaddad et al., 2013).

Several statistics have been suggested to measure LD (Hill and Weir, 1994; Terwilliger, 1995; Zhao et al., 2005; Gianola et al., 2013). Evaluation of these methods has shown that *r* ^2^ is less affected by allelic frequency and sample size than D' (Pritchard & Przeworski, 2001; Sved, 2009; Bohmanova et al., 2010). Even when the level of LD of populations is similar, this may still be the result of different evolutionary histories. In this regard, determining patterns of the persistency of LD phase (PLDP) is useful for genetic studies (Pritchard et al., 2000). A SNP in LD with quantitative trait loci may have one marker allele in phase with the beneficial allele for the trait in one breed, while in another breed, the phase may be different. Therefore, GS based on marker information in one population may not lead to genetic progress in another (De Roos et al., 2008). PLDP represents the amount of LD that is maintained between populations and is dependent on the divergence time of the breeds (Badke et al., 2012; Wang et al., 2013). Higher values of PLDP between populations indicate more ancestral LD in common, such that the genomic information can be more reliably inferred between them (Mokry et al., 2014). PLDP can also be used to evaluate the relationships among populations, with those having a common history showing higher PLDP (Wang et al., 2013).

LD provides information to identify the genes and variations affecting quantitative traits in genome-wide association studies (GWAS) by inferring the distribution of recombination events. LD can also be used to evaluate diversity and population structure and to identify genomic regions affected by selection (Mokry et al., 2014). The pattern of LD can reveal the genetic history and the previous demography of a population and can be used to infer the effective population size (Ne) (Qanbari, 2020). Effective population size, Ne, is considered to be one of the most important parameters in population genetics and reflects the amount of genetic diversity, inbreeding, and genetic drift in the population (Frankham, 2005; Tenesa et al., 2007). A low value of Ne indicates limited genetic diversity in a population and affects the amount of genetic progress that can be made in breeding programs (Hayes et al., 2003). Ne can be determined by assessing the amount of LD at various distances along the genome (Sved, 1971; Hayes et al., 2003). High LD at long recombination distances reflects low Ne in recent generations (Hayes et al., 2003).

Buffaloes were introduced into Egypt from India, Iran, and Iraq during the seventh B.C. (Minervino et al., 2020). The three breeds from Iran are reared in three different geographical areas with completely different climatic conditions. The Azeri breed is mainly reared in the north-west and north of Iran (West Azerbaijan, East Azerbaijan province, Ardebil, and eastern parts of Gilan provinces), which have cold, sub-zero winters with heavy snowfall and hot, dry summers with temperatures reaching 35 C, the Khuzestani breed is found in the southwest (mainly in Khuzestan province), which has very hot and occasionally humid summers, with temperatures routinely exceeding 45°C degrees, while in the winter, it can drop below freezing, and the Mazandarani breed is reared along the coast of the Caspian Sea in the Mazandaran and Golestan provinces, which have a moderate climate with occasional humidity all around the year (Mokhber et al., 2019a). The Anatolian water buffalo is widespread in Northwestern Turkey, especially along the coast of the Black Sea, the middle of Anatolia, and also in Eastern Anatolia (Soysal et al., 2007). The Egyptian buffaloes are spread along the River Nile, in the Delta Region, and at the Fayum Oasis. With more than three million head, buffalo is the most important livestock species for milk production in Egypt. The Nili-Ravi breed is the most important livestock breed in Pakistan with more than 10 million head in Punjab, while the Kundi, with more than five million head, is the second most important breed in Pakistan (Minervino et al., 2020).

The present study describes genetic diversity, LD between adjacent SNPs, the trend of LD with increasing distance, and the patterns of PLDP and Ne using genomic data from buffalo breeds of Turkey, Egypt, Pakistan, and Iran, which are genetically closer together than other water buffaloes across the world (Colli et al., 2018).

## Materials and Method

### Genotype Determination and Data Edition

The present study used data for 83 water buffaloes, including 14 Azeri (AZI), 11 Khuzestani (KHU), and eight Mazandarani (MAZ) from Iran, 12 Anatolian buffalo (ANA) from Turkey, nine Kundi (KUN), and 14 Nili-Ravi (NIL) from Pakistan, and 15 Egyptian buffalo (EGY) to assess LD structure and calculate Ne (Table 1).

**TABLE 1:** Descriptive statistics for the studied buffalo populations.

Row	Population name	Population label	Country	Region	N before QC	Number after QC	SNP number after separating QC	SNP number after mergence
1	Azeri	AZI	Iran	Urmia, West Azerbaijan Province	14	14	66,989	57,455
2	Khuzestani	KHU	Iran	Ahvaz, Khuzestan Province	11	11	66,145	57,455
3	Mazandarani	MAZ	Iran	Miankaleh peninsula, Mazandaran Province	8	8	67,900	57,455
4	Anatolian	ANA	Turkey	Istanbul, Afyonkarahisar (western Anatolia) and Tokat (central Anatolia) Provinces	15	12	66,692	57,455
5	Egyptian	EGY	Egypt	-	16	15	66,145	57,455
6	Kundhi	KUN	Pakistan	-	10	9	69,451	57,455
7	Nili-Ravi	NIR	Pakistan	-	15	14	69,820	57,455
Total					89	83	82,043	57,455

The samples were genotyped using the Axiom® Buffalo Genotyping 90 K array (Affymetrix, Santa Clara, CA, United States) that were mapped to the bovine sequence (UMD3.1 Bos Taurus) (Iamartino et al., 2017). Details on the animals and the genomic data are presented in Table 1. The genotype data were edited with Plink software (Purcell et al., 2007), and animals and loci with more than 5% missing genotypes (CR_IND_ and CR_SNP_), monomorphic genotypes, and genotypes with minor allele frequency (MAF) less than 5% were eliminated. MAF and missing genotypes of individuals and SNPs were filtered separately for each genotypic group. Then, the genomic data of all genetic groups were integrated, and the common genetic markers were identified. Finally, the SNPs that were not in the Hardy-Weinberg equilibrium were excluded, and the missing genotypes were imputed using BEAGLE software (Browning & Browning, 2007).

### Assessment of Population Structure

Discriminant analysis principal component (DAPC), principal component analysis (PCA), Weir and Cockerham unbiased fixation index (F_ST_), and population admixture were used to obtain a general overview of the structure of each population and identify animals falling outside their breed group. DAPC, PCA, and F_ST_ were performed using the adegenet package (Jombart and Ahmed, 2011), GeneABEL software (Price et al., 2006), and R scripts using R software (http://www.rproject.org/), respectively. Additionally, the genetic structure of the populations was evaluated using ADMIXTURE software (Alexander et al., 2009).

### LD Analysis

After determining the population structure of each genetic group, the patterns of LD were estimated. The values of LD between adjacent SNP as well as paired bases at distances of 0–15 Mb were obtained in each population and evaluated using the statistics *r* ^2^ (Hill and Robertson, 1968) and D′, which were calculated as follows:

$$r^{2}=\frac{{\left(D\right)}^{2}}{\left(freq\;A*freq\;a*freq\;B*frq\;b\right)},$$

where

D=freq AB−freq A∗freq B

and

$$D^{\prime }=\{\begin{array}{c} \frac{D}{\min\left(freq\;A*freq\;b,freq\;B*freq\;a\right)}if\;D>0\\ \frac{D}{\min\left(freq\;A*freq\;B*freq\;a*freq\;b\right)}if\;D<0 \end{array},$$

where SNP pairs had alleles *A* and *a* at the first locus and *B* and *b* at the second locus, *freq A*, *freq a*, *freq B*, and *freq b* denote frequencies of alleles *A, a, B*, and *b*, respectively, and *freq AB* denotes frequency of the haplotype *AB* in the population.

The *r* ^2^ statistic represents the correlation between alleles at two loci and is less dependent on allele frequencies in finite population sizes compared with other LD measures (Lewontin, 1988; Abecasis et al., 2001; Mueller, 2004) and is the preferred measure for biallelic markers (Zhao et al., 2007). Therefore, *r* ^2^ was used in the Ne, LD decay, and PLDP analyses. The *r* ^2^ statistic is biased by sample size, and this bias is higher for a smaller sample size. Correction methods discussed by Hui and Burt (2020), Waples et al. (2016), Villa-Angulo et al. (2009), Weir and Hill (1980), and Sved (1971) were applied to the estimate of *r* ^2^ in this study. Due to the small sample size for each population, the information was corrected for the sample number and uncertainty of the gametic phase using the following equation (Weir and Hill, 1980; Corbin et al., 2012), which was implemented in SNeP software (Barbato et al., 2015).

$$r_{adj}^{2}=\;r^{2}-{\left(\beta n\right)}^{-1},$$

where *n* is the number of individuals sampled, *β* = 2 when the gametic phase is known, and *β* = 1 if instead the phase is not known (Weir and Hill, 1980).

To determine LD decay, paired markers that were common to all populations were grouped at distances between 0 and 15 Mb at 100 Kb intervals, and the mean *r* ^2^ was calculated for each group. The PLDP between populations was expressed as the correlation between the roots of the *r* ^2^ calculated for adjacent markers using the formula provided by Badke et al. (2012).

$$r_{ij}=\frac{\sum_{\left(i,j\right)}\left(r_{ij\left(A\right)}-{\bar{r}}_{A}\right)\left(r_{ij\left(B\right)}-{\bar{r}}_{B}\right)}{S_{A}S_{B}},$$

where *r*_*ij*_ is the correlation of phase between *r*_*ij(A)*_ in population A and *r*_*ij(B)*_ in population B, *S*_*A*_ and *S*_*B*_ are the standard deviation of *r*_*ij(A)*_ and *r*_*ij(B)*_, respectively, and *r*_*A*_ and *r*_*B*_ are the average *r*_*ij*_ across all SNP *i* and *j* within the common set of markers.

### Effective Population Size (Ne)

The corrected LD for each population was used to calculate Ne by applying the formula of $Ne=\left(\frac{1}{4c}\right)\left(\frac{1}{r^{2}}-1\right)$ (Sved, 1971), where Ne represents the effective population size of generation T, *r* ^2^ indicates the mean of LD for a given distance, and *c* is the distance between markers in Morgan (1 centimorgan was considered to be approximately equal to one megabase pair, Tenesa et al., 2007; Villa-Angulo et al., 2009). Generation was calculated to determine Ne (T) based genomic distance using the formula of T = 1/2c (Hayes et al., 2003).

## Results and Discussion

### Quality of Data

Before frequency and genotyping pruning, there were 89,988 SNPs and 89 individuals. In the first step, six individuals were removed for low genotyping success (MIND >0.05), 637 markers were excluded based on HWE (p≤5.7e-007), and 7,618 SNPs for missing information (GENO >0.05). A total of 83 individuals with 82,043 SNPs passed the first step of QC; the total genotyping rate of these remaining individuals was 0.985. In the second step, MAF was assessed in each population separately, and SNPs with MAF>0.05 were removed (Table 1). Then, the populations were merged to create a common dataset of 57,426 SNP markers with MAF higher than 0.05 for each population that passed all the filters. These were used in subsequent analyses in snppLD software (Sargolzaei M, University of Guelph, Canada). These markers covered 2,646.07 Mb of the bovine genome. The mean distance between these markers was 46.07 Kb, and minimum and maximum distances were 42.4 Kb on chromosome BTA 24 and 68.2 Kb on the BTA X, respectively.

### Assessment of Population Structure

**FIGURE 1:**
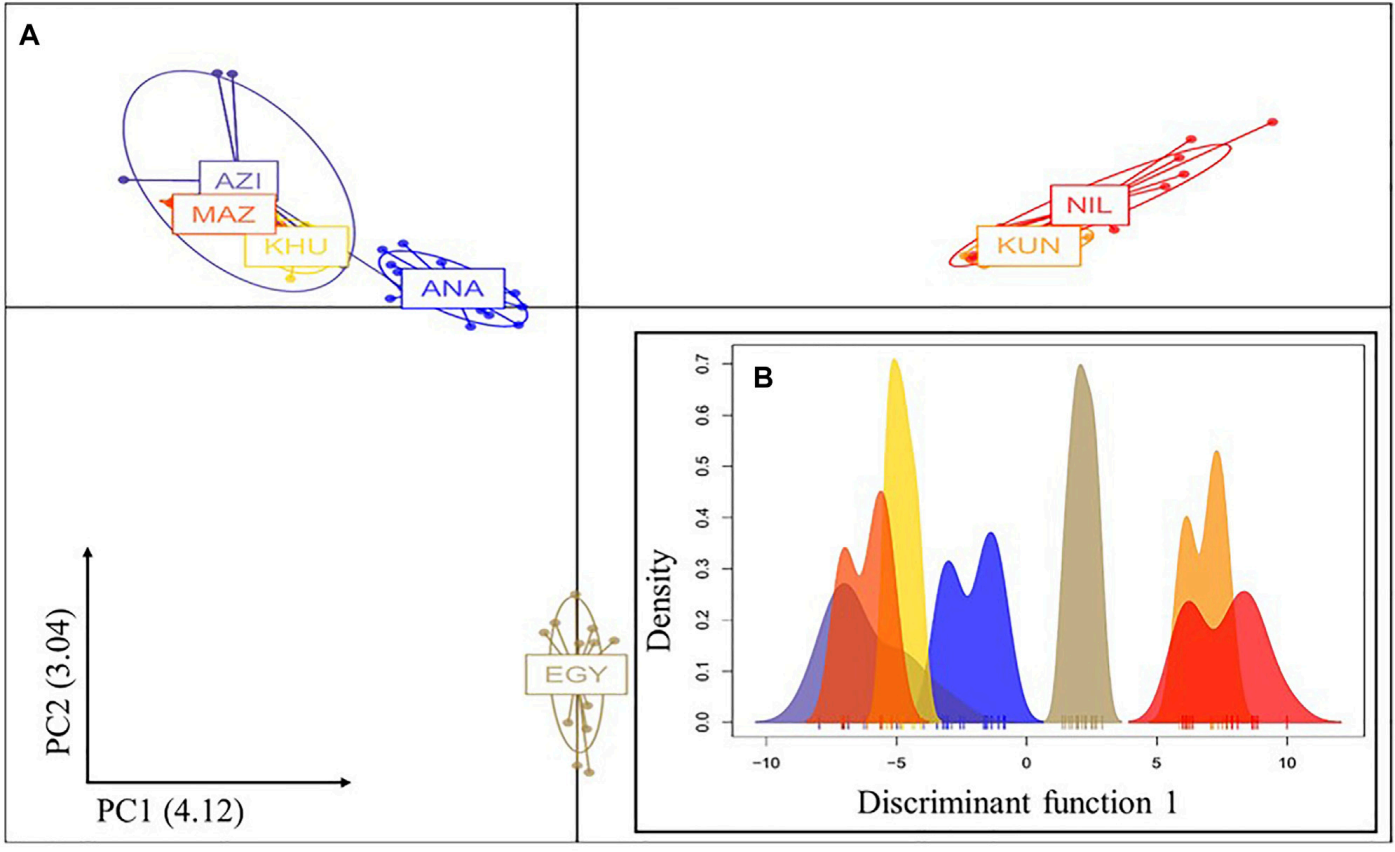
A two first PCs and B first PC only of the DAPC analysis of the water buffalo populations studied. Clusters are indicated by different colors (blue, gray, and green), and populations are identified by their abbreviations AZI, KHU, MAZ, ANA, EGY, KUN, and NIL.

Understanding of population genetic structure is important to assess population stratification for GWAS, breeding program design, and developing strategies for genetic resources preservation. DAPC, PCA, and admixture analysis results were used to assess population structure. Both PCA and DAPC methods gave similar results. In both methods, genotype data formed three distinct clusters in the first two PCs. The ANA population from Turkey was partially separated from the Iranian cluster, which includes AZI, KHU, and MAZ (Figure 1 and Supplementary Figure S1). The first two PCs in the DAPC accounted for 7.16% of the total variance, 4.12% in the first, and 3.04% in the second dimension (Figure 1). The first 10 PCs of DAPC only accounted for about 24% of the total variance (Supplementary Figure S2). In the PCA analysis, the first and second PCs explained 4% and 2% of the variance, respectively (Supplementary Figure S1). The ANA along with AZI, KHU, and MAZ formed overlapping groups with the AZI buffalo being interspersed among the KHU, MAZ, and ANA populations (Figure 1A,B). The EGY and populations from Pakistan (KUN and NIL) formed two additional distinct clusters (Figure 1). The geographic proximity of Iranian populations with the ANA in Turkey makes gene flow between these two populations likely, which would reduce the differentiation between them. In the analysis of Colli et al. (2018), the populations assessed in the present study belonged to one cluster, which is because these populations are genetically similar when compared with other more genetically distinct breeds worldwide. The results presented here are consistent with other studies focused on Iranian buffaloes where no differences (Strillacci et al., 2021) or very small genetic differentiation was observed (Rahmaninia et al., 2015; Azizi et al., 2016; Mokhber et al., 2018; Ghoreishifar et al., 2020).

There were small differences in F_ST_ among the studied populations (Supplementary Table 1); in most cases, the difference between pairs of populations was less than 0.05, indicating low genetic differentiation according to Wright’s classification. The reason for this is because there was high within, compared with between-population variance. However, the F_ST_ results confirmed the DPCA and PCA analyses by separating the populations into three genetic groups. The mean F_ST_ value across populations was 0.045 and varied from 0.011 for AZI from Iran and ANA from Turkey to 0.077 for MAZ from Iran and KUN from Pakistani. The smallest genetic distance was between the Iranian buffaloes and ANA from Turkey, while the largest distance was between the Iranian buffalo and KUN and NIL from Pakistani.

**FIGURE 2:**
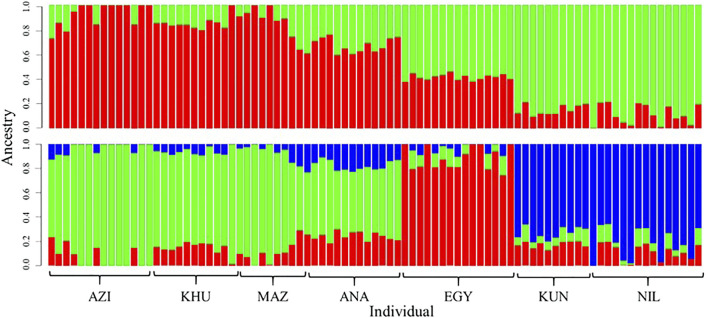
Genetic composition of buffalo breeds revealed with ADMIXTURE software at K = 2 **(top)** and K = 3 **(bottom)**. Individuals are represented with vertical colored bars. Genomic components are assigned different colors.

Population structures were investigated using ADMIXTURE software, assuming K as ancestral populations ranging from one to seven. Based on cross-validation error criteria, K = 2 and three had suitable resolution (Figure 2). The first subdivision at K = 2 distinguished between Pakistani (KUN and NIL) and the others populations (AZI, KHU, MAZ, ANA, and EGY) (Figure 2). At K = 3, the EGY population becomes genetically distinct, giving three groups that coincide with DAPC and PCA clusters. The ADMIXTURE analysis shows that there are genetic components shared among all the populations explaining the overlap between clusters.

### LD Analysis

We calculated both *r* ^2^ and D′ for adjacent SNPs in the populations for each chromosome (see S1 Supplementary Table S1). Because of the small sample size, uncorrected LD values were similar among breeds within clusters, in particular the Iranian breeds, AZI, KHU, and MAZ and Pakistani breeds, KUN and NIL. Results were also corrected for sample size. The values of corrected *r* ^2^ for the pooled Iranian breeds (IRI), ANA, EGY, and PAK populations were 0.24, 0.28, 0.27, and 0.22, respectively (Table 2). At the chromosome level, chromosomes 25 of the PAK population and chromosomes X of the ANA had the maximum corrected *r* ^2^ values, respectively (Table 2 and Supplementary Table S2). Previous studies reported that a small sample size (less than 25) leads to an overestimate of *r* ^2^ (Khatkar et al., 2008; Deng et al., 2019), while Bohmanova et al. (2010) reported that at least 55 and 444 individuals were required for accurate estimation of *r* ^2^ and D′, respectively. Other studies have found that D′ statistics are more affected by population size than *r* ^2^ (Ardlie et al., 2002; Jemaa et al., 2019). Therefore, estimated *r* ^2^ values in the present study are more reliable than the D’ statistics. Comparing uncorrected and corrected *r* ^2^ for sample size revealed that the differences in smaller populations are greater. The corrected vs. uncorrected *r* ^2^ values changed from 0.27 to 0.24 (around 0.02 units) in the pooled IRI, which has 33 individuals, but from 0.35 to 0.28 (around 0.07 units) in ANA with 12 individuals, 0.34 to 0.27 (around 0.07 units) in EGY with 15 individuals, and 0.27 to 0.22 (around 0.05 units) in PAK with 23 individuals (Supplementary Table S2). If Iranian and Pakistani populations were considered individually, the bias in *r* ^2^ estimates increased because of the smaller sample size in the individual populations. These results show that correction of *r* ^2^ for sample size is necessary.

**TABLE 2:** Distance and linkage disequilibrium (corrected *r* ^2^) between adjacent polymorphic SNPs for IRI, ANA, EGY, and PAK water buffalo populations.

Chromosome	SNP number	Distance (Kb)	IRI	ANA	EGY	PAK
1	3,583	44.1	0.24 ± 0.25	0.27 ± 0.26	0.28 ± 0.27	0.23 ± 0.24
2	3,024	45.1	0.24 ± 0.26	0.3 ± 0.28	0.27 ± 0.27	0.24 ± 0.24
3	2,708	44.8	0.23 ± 0.25	0.27 ± 0.27	0.28 ± 0.27	0.22 ± 0.23
4	2,731	44.1	0.23 ± 0.24	0.28 ± 0.27	0.28 ± 0.27	0.22 ± 0.22
5	2,601	46.3	0.24 ± 0.26	0.29 ± 0.27	0.29 ± 0.27	0.24 ± 0.24
6	2,649	45	0.23 ± 0.25	0.29 ± 0.28	0.26 ± 0.26	0.21 ± 0.22
7	2,505	44.9	0.22 ± 0.24	0.26 ± 0.27	0.25 ± 0.26	0.22 ± 0.23
8	2,416	46.8	0.24 ± 0.26	0.28 ± 0.27	0.28 ± 0.27	0.23 ± 0.23
9	2,268	46.4	0.22 ± 0.24	0.3 ± 0.28	0.26 ± 0.26	0.23 ± 0.23
10	2,307	45	0.22 ± 0.24	0.28 ± 0.26	0.28 ± 0.27	0.22 ± 0.23
11	2,368	45.2	0.23 ± 0.25	0.31 ± 0.28	0.27 ± 0.26	0.23 ± 0.23
12	1,933	47.1	0.22 ± 0.25	0.27 ± 0.26	0.26 ± 0.26	0.22 ± 0.23
13	1,872	44.7	0.21 ± 0.23	0.23 ± 0.24	0.26 ± 0.26	0.2 ± 0.22
14	1,945	42.7	0.22 ± 0.24	0.25 ± 0.25	0.26 ± 0.26	0.21 ± 0.22
15	1,798	47.2	0.19 ± 0.24	0.29 ± 0.28	0.27 ± 0.27	0.2 ± 0.21
16	1,742	46.6	0.23 ± 0.26	0.27 ± 0.26	0.26 ± 0.27	0.24 ± 0.24
17	1,658	45.1	0.23 ± 0.26	0.29 ± 0.28	0.25 ± 0.25	0.22 ± 0.23
18	1,397	47	0.2 ± 0.23	0.25 ± 0.26	0.24 ± 0.26	0.19 ± 0.21
19	1,384	45.9	0.22 ± 0.25	0.27 ± 0.26	0.25 ± 0.25	0.22 ± 0.23
20	1,584	45.3	0.2 ± 0.25	0.28 ± 0.27	0.28 ± 0.28	0.22 ± 0.23
21	1,510	45.7	0.22 ± 0.24	0.29 ± 0.28	0.22 ± 0.24	0.21 ± 0.22
22	1,379	44.4	0.22 ± 0.24	0.23 ± 0.24	0.25 ± 0.26	0.21 ± 0.23
23	1,115	46.7	0.22 ± 0.25	0.28 ± 0.28	0.27 ± 0.27	0.21 ± 0.22
24	1,462	42.4	0.21 ± 0.22	0.28 ± 0.27	0.26 ± 0.26	0.21 ± 0.22
25	991	43.1	0.2 ± 0.24	0.26 ± 0.26	0.24 ± 0.26	0.18 ± 0.21
26	1,178	43.5	0.2 ± 0.23	0.26 ± 0.26	0.24 ± 0.26	0.19 ± 0.21
27	1,017	44.6	0.2 ± 0.22	0.25 ± 0.25	0.24 ± 0.26	0.21 ± 0.22
28	1,043	44.1	0.22 ± 0.24	0.24 ± 0.25	0.27 ± 0.27	0.22 ± 0.23
29	1,076	47.2	0.2 ± 0.23	0.25 ± 0.26	0.24 ± 0.25	0.19 ± 0.21
30	2,181	68.2	0.29 ± 0.3	0.59 ± 0.32	0.39 ± 0.31	0.3 ± 0.3
Average	1914	46.7	0.24 ± 0.24	0.28 ± 0.27	0.27 ± 0.26	0.22 ± 0.29

The corrected average *r* ^2^ values for individual populations from Iran, including AZI, KHU, and MAZ, were consistent and slightly lower than the values reported by Mokhber et al. (2019a) for AZI and KHU but not for MAZ. They found an *r* ^2^ of 0.27, 0.29, and 0.32 for AZI, KHU, and MAZ, respectively, using a larger dataset for AZI and KHU, but not MAZ. The difference in *r* ^2^ for MAZ was due to the correction method for average *r* ^2^ values.

Much lower values that obtaining in the present study were obtained *r* ^2^ values were obtained using the 90 K Buffalo SNP genotyping array in a study of 430 pure Mediterranean buffaloes and 65 Chinese crossbred buffalo, which gave an *r* ^2^ of 0.13 and 0.09, respectively (Deng et al., 2019). The mean value *r* ^2^ for adjacent SNPs in a study of 384 Brazilian Murrah buffaloes using the Bovine HD array in buffalo (Borquis et al., 2014), which provided 16,580 polymorphic loci from the 688,593 markers on the array, obtained and *r* ^2^ of 0.29. When the 90 K Buffalo Axiom array was used with a sample of 452 Brazilian Murrah buffaloes, 58,585 SNPs were polymorphic, and the same genome-wide *r* ^2^ of 0.29 was obtained, while the *r* ^2^ and |D|' for each chromosome were between 0.17 and 0.33 and 0.41 and 0.80, respectively (Cardoso et al., 2015). Using genomic information for 70 Iranian native cattle belonging to seven breeds (10 samples for each breed), Karimi et al. (2015) obtained average *r* ^2^ for the adjacent SNP markers of between 0.321 and 0.393.

The percentages of adjacent markers in IRI, ANA, EGY, and PAK populations with corrected *r* ^2^ greater than 0.2 (0.12) were 46, 52, 51, and 47% (Supplementary Table S3). The mean *r* ^2^ for adjacent markers can be used to assess their suitability for GWAS and the estimation of breeding values. An *r* ^2^ higher than 0.3 is recommended for GWAS (Ardlie et al., 2002), while an LD of more than 0.2 is considered essential for estimating genomic breeding values (Meuwissen et al., 2001).

The mean and standard deviation of D′, which represents the frequency of recombination events between adjacent SNPs, was 0.74, 0.67, 0.64, and 0.72 for IRI, ANA, EGY, and PAK, respectively (see Supplementary Table S2). A D′ value close to one implies that ancestral haplotypes have not been separated by recombination over time. In general, D′ is more affected by sample size than *r* ^2^ but less influenced by allele frequency. The pooled Iranian (IRI) population had the highest D' (0.74), while the EGY had the lowest (0.64).

Population history, including mutation, selection, recombination, and migration, affects the genome structure and will be reflected in the value of *r* ^2^. Factors such as sample size, the threshold for the frequency of rare alleles, the density of SNP, and the distances between markers will also affect the results. Further, the way that samples are selected may distort the diversity estimated for a population. A study on pig breeds using a 50 K SNP array and a large number of samples in each genetic group identified high selection pressure and low diversity in populations as the reasons for the high LD found (Badke et al., 2012). In the present study, we pooled some populations because of the small sample size; in addition, we corrected LD estimates for sample size, and only SNPs with reasonable MAF (>0.05) were included. Because D’ is more sensitive to sample size, we used the corrected *r* ^2^ values for subsequent analysis of LD decay, PLDP, and Ne.

### LD Decay

**FIGURE 3:**
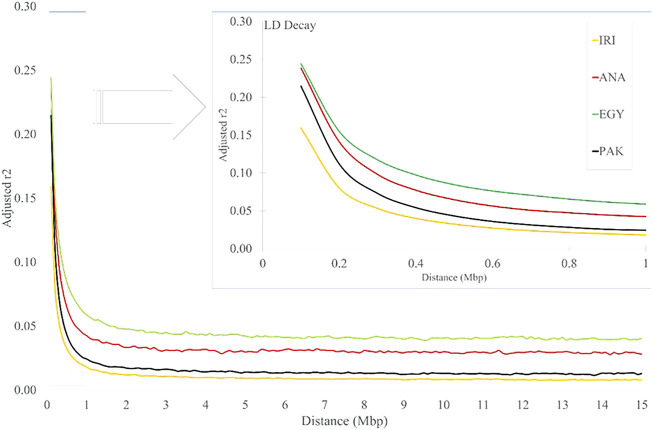
LD decay for increasing distance (Mb) for IRI, ANA, EGY, and PAK water buffalo populations.

As expected, the average *r* ^2^ values decreased with increasing distance between pairwise SNPs for all the studied populations (Figure 3 and Supplementary Table S4). The values for IRI, ANA, EGY, and PAK were 0.367, 0.441, 0.411, and 0.432, respectively, for distances less than 10 Kb and 0.16, 0.24, 0.24, and 0.21, respectively, for distances less than 100Kb, which reduced rapidly to 0.018, 0.042, 0.059 and 0.024 (respectively) for a distance between markers of 1 Mb (Figure 3 and Supplementary Table S4). In all the populations, the LD then remained constant for distances greater than 2 Mb to the longest distance considered (15 Mb) (Supplementary Table S4). The LD decayed slowly in EGY and ANA and in individual Iranian and Pakistani breeds. The highest LD, especially at longer distances, was seen MAZ and KUN. This may be due to the rapid decline of these populations in more recent generations. The effect of correcting *r* ^2^ was smaller (6–20 percent) for distances <10 kb and increased to more than 50 percent for distances >1 Mb and to 70–80 percent for distances >10 Mb. This suggests that *r* ^2^ values are more affected at longer distances by population size (Supplementary Table S4). Comparing the LD for individual Iran populations (AZI, KHU, and MAZ) obtained here with Mokhber et al. (2019a), which used a larger sample size (more than 200), LD estimates at >100 Kb were similar, whereas at greater distances, the results were significantly different.

Lu et al. (2020) calculated the rate of LD decay in Chinese river and swamp buffaloes and found that the LD of river buffaloes was higher than that of a swamp and that the rate of LD decay in swamp buffaloes was higher than for river buffaloes for all marker distances. These data reflect the stronger genetic selection in the river buffalo breeds compared with the swamp breeds. The rate of LD decay in Chinese crossbred buffaloes has been reported to be higher than in pure Mediterranean buffalo at a distance of 600 Kb (Deng et al., 2019), possibly as a result of recent cross-breeding.

A similar situation is seen for cattle where the LD is higher in dairy cattle, which are under stronger selection than beef breeds (Qanbari et al., 2010). The pattern of LD in German Holstein cattle gave an *r* ^2^ of about 0.3 for a distance less than 25Kb, which decreased to 0.24 for distances of 50–75 Kb (Qanbari et al., 2010), whereas in Australian Holstein bulls, *r* ^2^ varied from 0.402 to 0.073 as the distance increased from 20 to 500 Kb (Khatkar et al., 2008). For beef cattle, where selection is less intense, the *r* ^2^ for Angus, Charolais, and crossbred beef breeds (Angus × Charolais) decreased from 0.23 to 0.19, 0.16 to 0.12, and 0.15 to 0.11, respectively, for distances 30 to 100Kb, respectively (Lu et al., 2012).

### Persistency of LD Phase

PLDP was calculated from the correlation between paired SNPs at distances of 0–15 Mb. An increase in the distance led to a decrease in PLDP between breeds (see Table 3 and Supplementary Table S4). At distances less than 100Kb, PLPD in all the populations was higher than 0.95 for buffalo populations from Iran, Turkey, Egypt, and Pakistan, which decreased to between 0.7 and 0.97 at 200Kb and then reduced rapidly. However, from 500 Kb to 1 Mb, the reduction in PLPD was less than seen between 200 and 500 Kb (Table 3 and Supplementary Table S5). The PLDP within breeds from the same geographical area that formed pools was higher than the other comparisons (Supplementary Table S5).

**TABLE 3:** Consistency of gametic phase at given distances between IRI, ANA, EGY, and PAK water buffalo populations.

Populations	Distances between paired SNPs (kbp)									
	>100	100–200	200–300	300–400	400–500	500–600	600–700	700–800	800–900	900–1,000
IRI-ANA	0.956	0.876	0.773	0.463	0.407	0.229	0.296	0.011	0.140	0.216
IRI_EGY	0.969	0.905	0.715	0.287	0.271	0.203	0.099	0.391	0.173	0.208
IRI_PAK	0.958	0.848	0.620	0.292	0.324	0.180	0.195	0.176	0.060	0.141
ANA-EGY	0.956	0.876	0.773	0.463	0.407	0.229	0.296	0.011	0.140	0.216
ANA_PAK	0.969	0.905	0.715	0.287	0.271	0.203	0.099	0.391	0.173	0.208
EGY_PAK	0.966	0.923	0.713	0.423	0.282	0.149	0.204	0.184	-0.059	0.145

PLPD among individual populations from Iran was above 0.95 for a distance less than 100Kb, which is similar results of Mokhber et al. (2019a) who reported values of 0.99, 0.96, and 0.95 at distances less than 100Kb, which reduced to 0.74, 0.25, and 0.12 at distances below than 1 Mb for AZI-KHU, AZI-MAZ, and KHU-MAZ populations, respectively.

These high PLPD values suggest that there may have been genetic exchange among these populations. The highest correlations previously reported among other pure and crossbred buffalo populations were 0.47 at the distance of 100 Kb (Deng et al., 2019), showing that the LD phase between independent populations tends not to be maintained. The value of PLDP among European, African, and African-European cattle breeds has been reported as 0.77, 0.71, and 0.65, respectively, at distances less than 10Kb and below 0.5 at distances greater than 50 Kb (Gautier et al., 2007). In Australian Holstein and New Zealand Jersey breeds, the PLDP correlation was 0.97 (De Roos et al., 2008), which is surprisingly high for breeds with different genetic histories. For beef breeds, PLDP between Charolais and Angus, Charolais and crossbred cattle, and Angus and Crosses was 0.84, 0.81, and 0.77, respectively, at distances less than 70 Kb (Lu et al., 2012), so that exchange of information among these populations should be treated with caution.

### Ne

**FIGURE 4:**
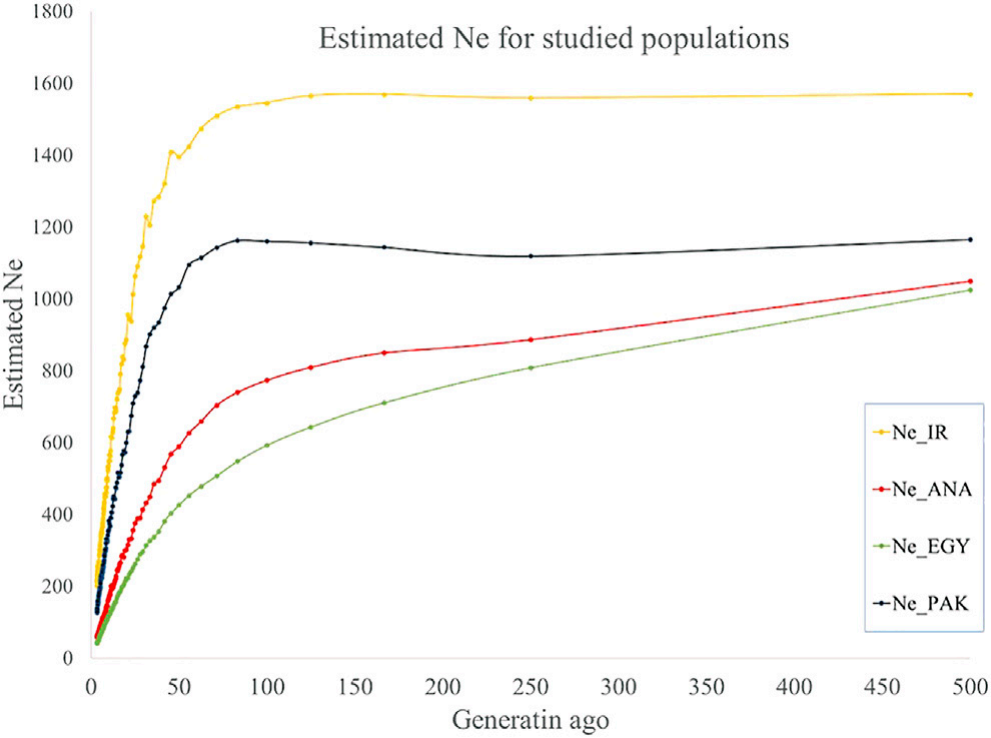
Estimated Ne for IRI (pooled Iranian breeds including AZI, KHU, and MAZ), ANA, EGY, and PAK (pooled Pakistani breeds including KHU and NIL) water buffalo populations for past generations.

Ne was estimated from the last 500 to recent generations in the present study. A trend of decreasing Ne was observed from more distant to recent generations: from 1,570 to 212, 1,049 to 59, 1,025 to 43, and 1,165 to 131 for IRI, ANA, EGY, and PAK breeds, respectively, from 500 generations ago to three last generations (Figure 4 and Supplementary Table S6). Similar trends for a decline in Ne from past to recent generations have been reported for buffalo (Mokhber et al., 2019b) other species (Sargolzaei et al., 2008; Moradi et al., 2012). The Ne of Canadian and American Holstein cattle decreased from 1,400 to less than 100 from 500 generations ago to recent generations (Sargolzaei et al., 2008). For sheep, the Ne of Zel and Lori-Bakhtiari breeds reduced from 4,900 to 840 and 4,900 to 532 animals from 2000 generations ago to the 20 last generations, respectively (Moradi et al., 2012). Ne for Sunite, German Mutton Merino, and Dorper sheep breeds has decreased from 1,506 to 207, 1,678 to 74, and 1,506 to 67, respectively, from 2000 generations ago to the seven last generations (Zhao et al., 2014).

The conservation of genetic and biological diversity is dependent on Ne (Wang, 2005). According to the FAO (1992), when Ne is equal to 25, 50, 125, 250, and 500, genetic diversity will shrink 18, 10, 4, 1.6, and 0.8 percent over 10 next generations, respectively. Evidence accumulated since 1980 shows that a Ne of more than 100 is necessary to maintain fitness over the subsequent 10 generations. Meuwissen (2009) showed that, with Ne greater than 100 individuals, the population would be sufficiently genetic diverse to survive in the long term, while to conserve the evolutionary potential of the population, it is better than Ne is more than 1,000 individuals (Frankham et al., 2014).

The present study showed that Ne of Iranian and Pakistani populations are greater than the population size threshold necessary to be genetically viable (Meuwissen, 2009). The main concern for all the studied populations is the rapid reduction in Ne in recent generations. Therefore, controlling the decline in Ne and increase in efficiency of economic production, e.g., by well-designed breeding programs, is necessary to prevent increasing inbreeding and eventually genetic extinction.

## Conclusion

In the present study, the LD structure, PLDP, and Ne were determined for seven buffalo populations and two populations pooled based on country or origin. The level of LD found indicated that it is appropriate to use the Affymetrix Axiom 90 K SNP genomic array for GWAS and GS in these populations. The correlation between the LD information and PLDP between geographically close populations was high, meaning that genomic information from one population can be used efficiently to predict genetic effects in another. We found that results are affected by sample size, which could be partially corrected for; however, additional data should be obtained to be confident of the results. The Ne in recent generations has declined to the extent that breeding plans are urgently required to ensure that these buffalo populations are not at risk of being lost.

## Data Availability

The original contributions presented in the study are included in the article/Supplementary Material; further inquiries can be directed to the corresponding author.

## Supplementary Material

Supplementary Figure S1 Principal components analysis based on the genomic kinship coefficients between all studied individuals.

Supplementary Figure S2 Principal components analysis based on the genomic kinship coefficients between all studied individuals.

Supplementary Figure S3 to 11 LD decay over physical distance for AZI, KHU, MAZ, IRI, ANA, EGY, KUN, NIL, and PAK, separated for each population and chromosomes.

Supplementary Table S1 Genetic diversity between all studied populations by unbiased F_ST_ statistics.

Supplementary Table S2 Table of mean| D'| and uncorrected and corrected *r* ^2^ values for AZI, KHU, MAZ, IRI, ANA, EGY, KUN, NIL, and PAK buffalo populations.

Supplementary Table S3 Frequency of *r* ^2^ and |D'| values for AZI, KHU, MAZ, IRI, ANA, EGY, KUN, NIL, and PAK buffalo populations.

Supplementary Table S4 Average LD decay over physical distance for AZI, KHU, MAZ, IRI, ANA, EGY, KUN, NIL, and PAK buffalo populations.

Supplementary Table S5 Consistency of gametic phase at given distances for AZI, KHU, MAZ, IRI, ANA, EGY, KUN, NIL, and PAK buffalo breed pairs.

Supplementary Table S6 Effective population size for AZI, KHU, MAZ, IRI, ANA, EGY, KUN, NIL, and PAK buffalo breed in a given number of generations ago.
