# Supplementary figures and images for: Linkage Disequilibrium and Effective Population Size of Buffalo Populations of Iran, Turkey, Pakistan, and Egypt Using a Medium Density SNP Array
Source: Front Genet. 2021 Dec 7;12:608186. doi: 10.3389/fgene.2021.608186 (PMC8689148; doi:10.3389/fgene.2021.608186)

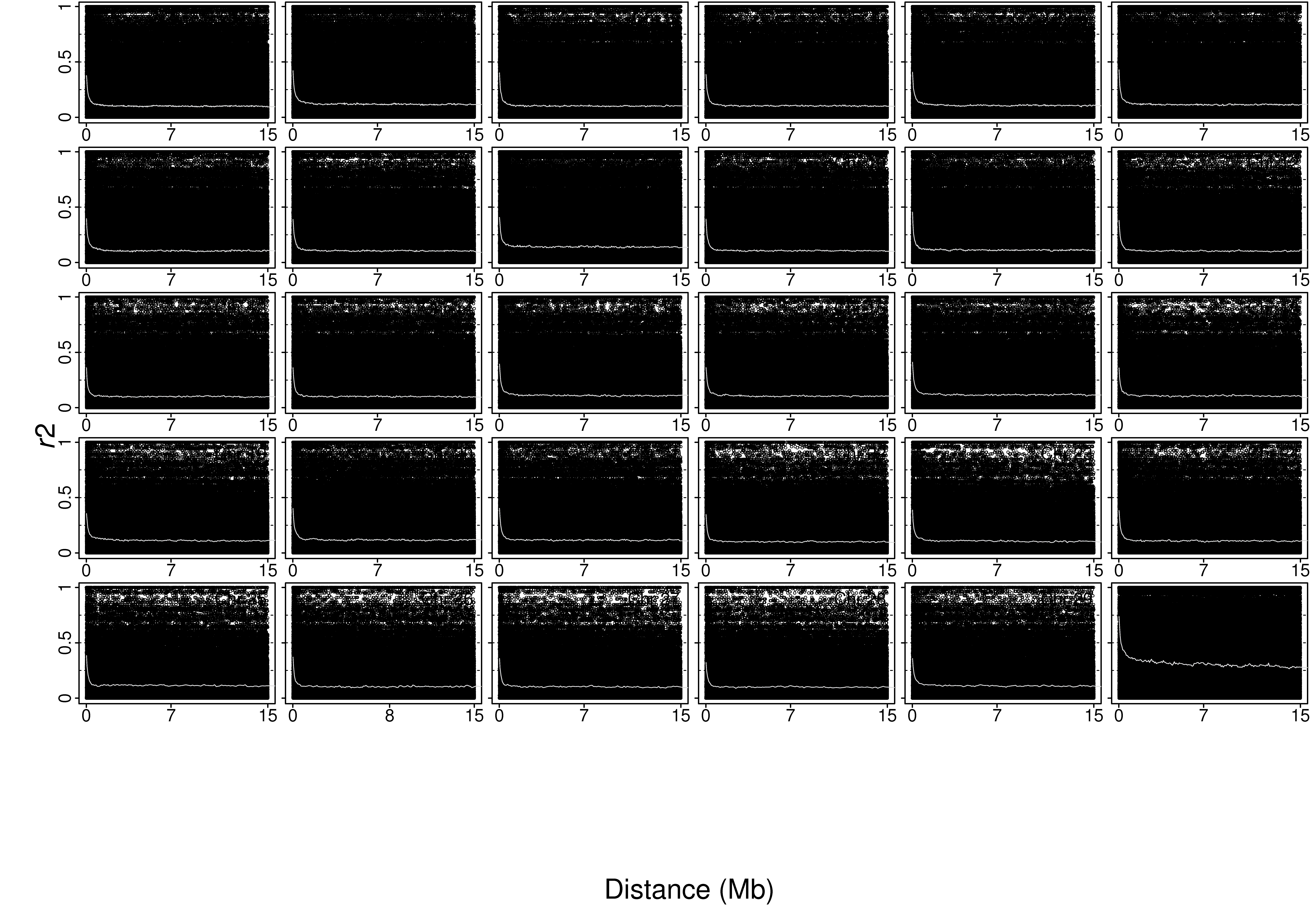

Supplement: Supplementary file 2 [file Image11.PNG]

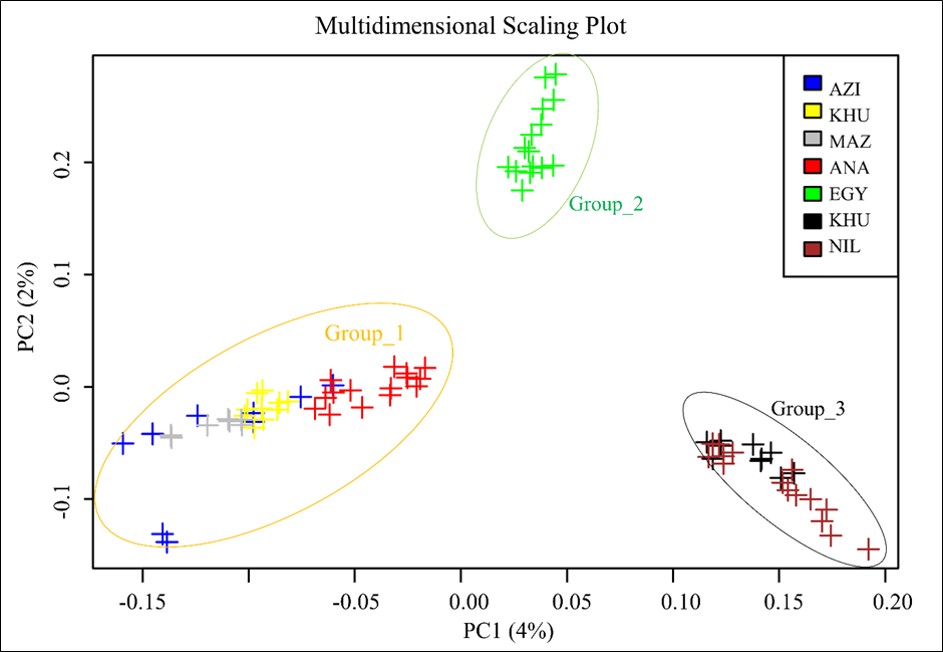

Supplement: Supplementary file 5 [file Image1.JPEG]

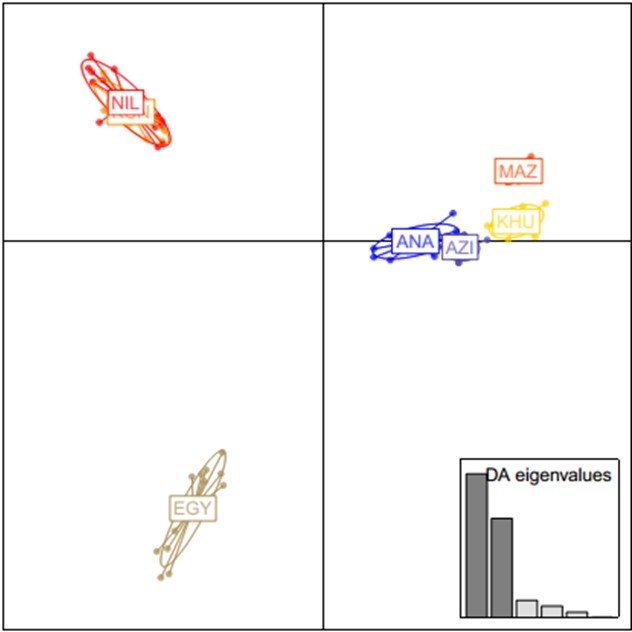

Supplement: Supplementary file 6 [file Image2.JPEG]

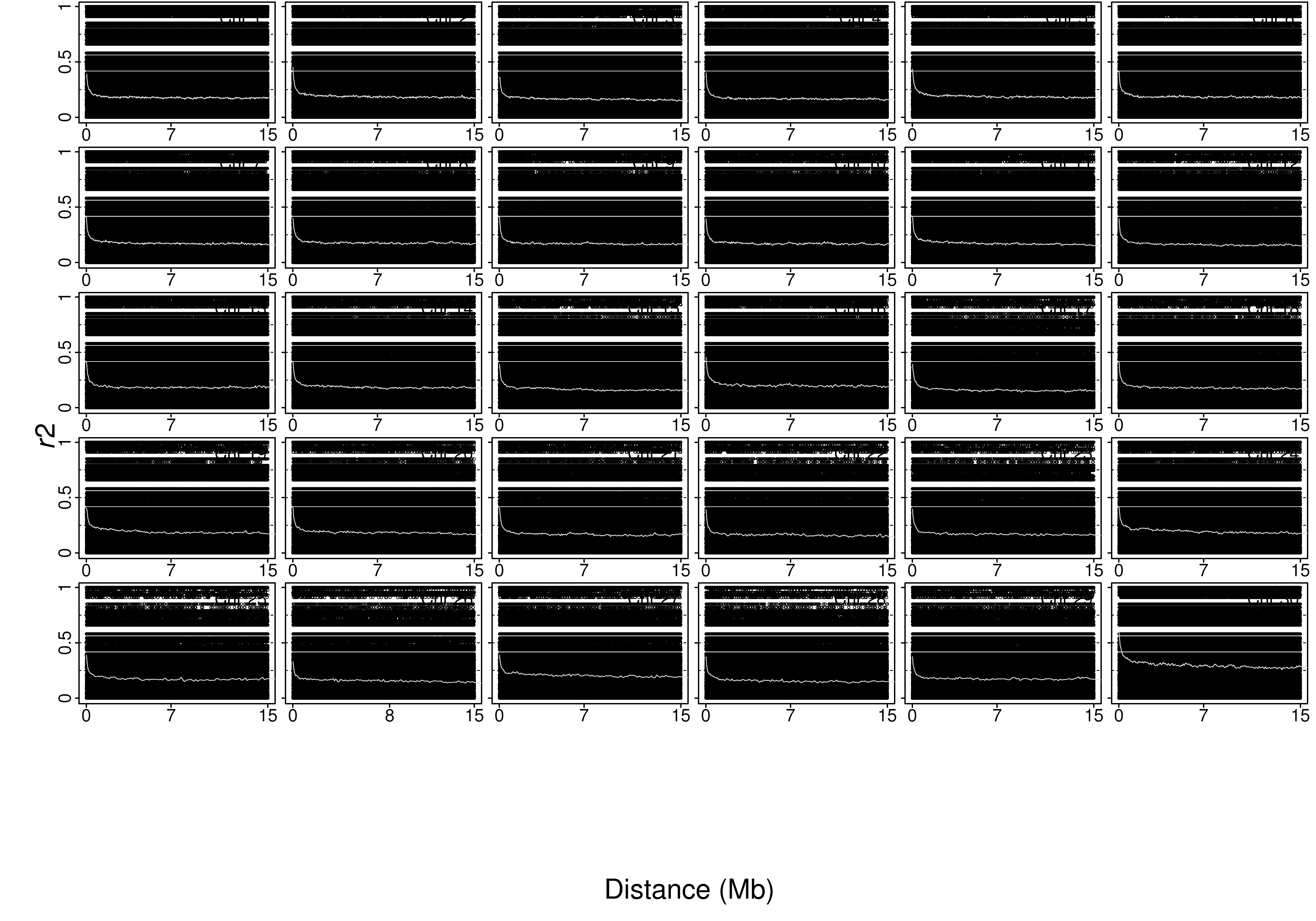

Supplement: Supplementary file 7 [file Image5.PNG]

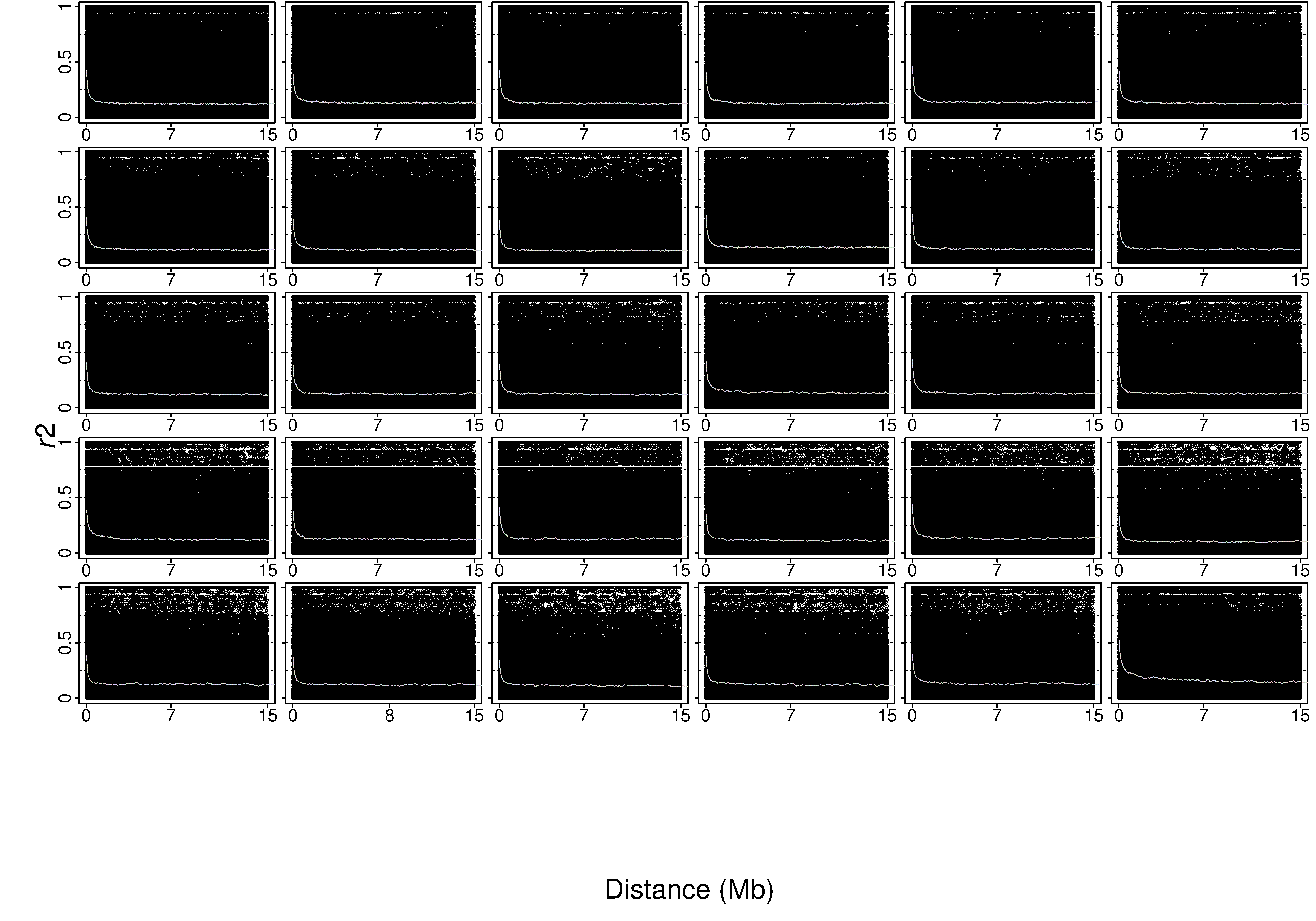

Supplement: Supplementary file 8 [file Image4.PNG]

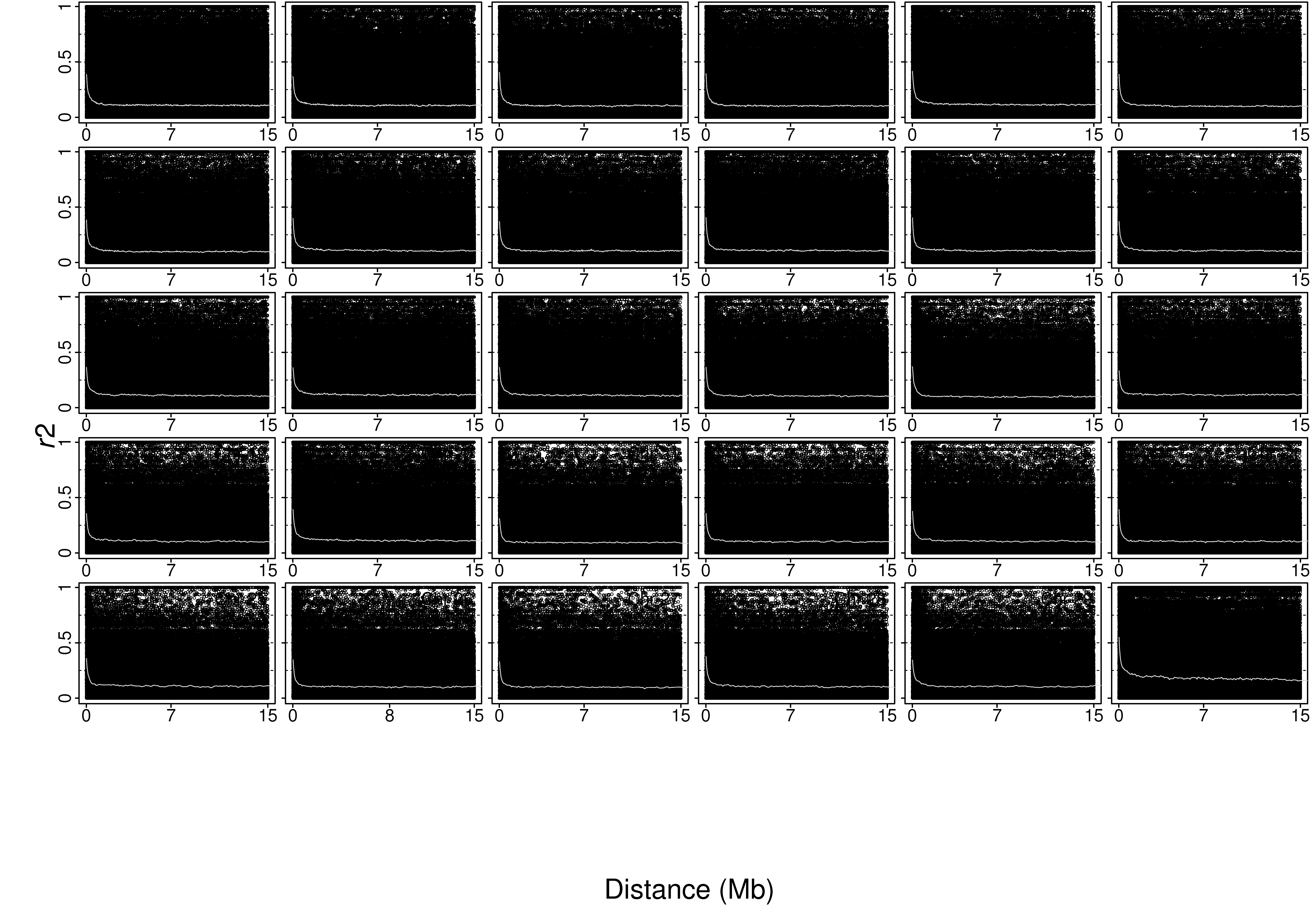

Supplement: Supplementary file 12 [file Image8.PNG]

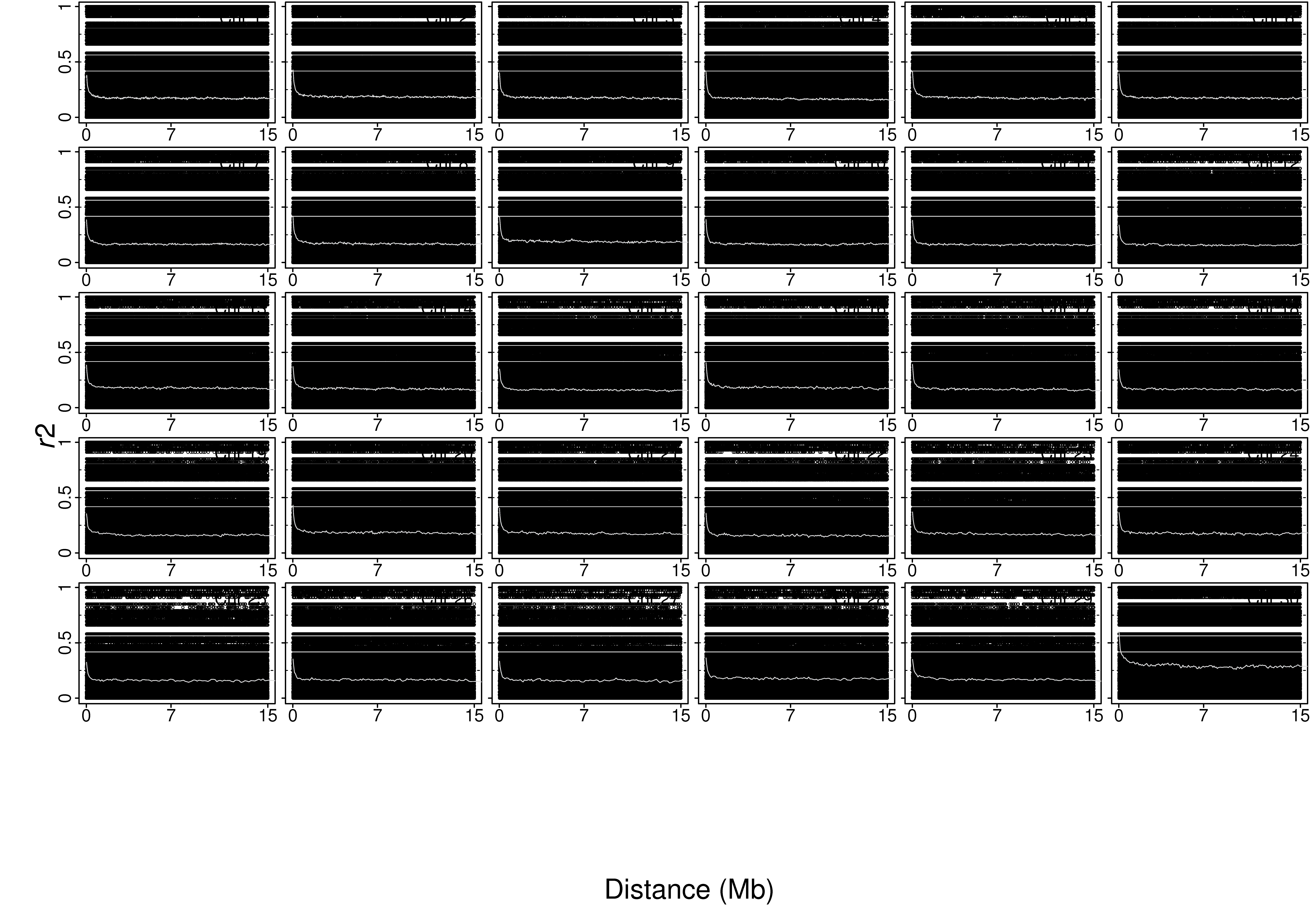

Supplement: Supplementary file 13 [file Image9.PNG]

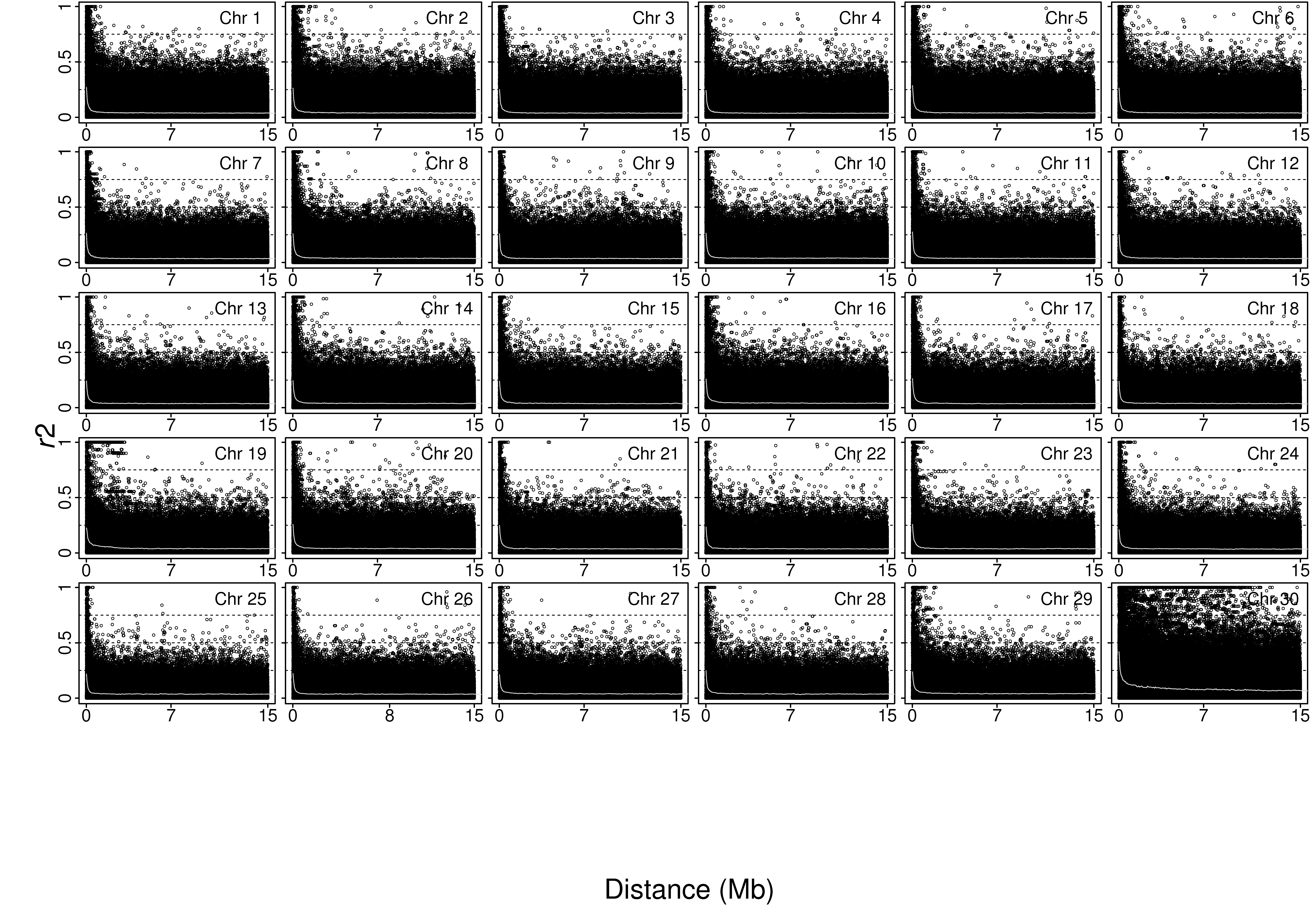

Supplement: Supplementary file 15 [file Image6.PNG]

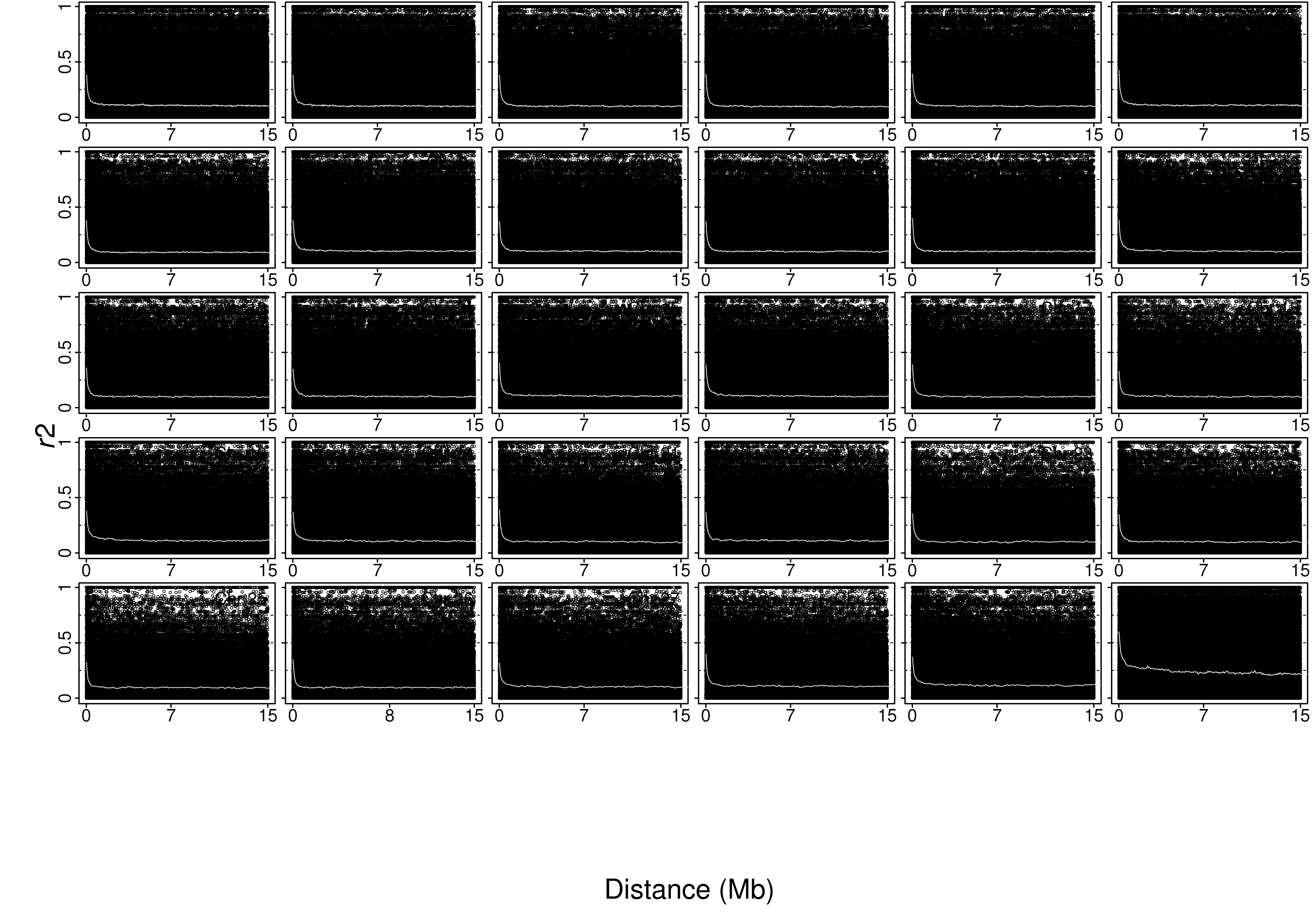

Supplement: Supplementary file 16 [file Image3.PNG]

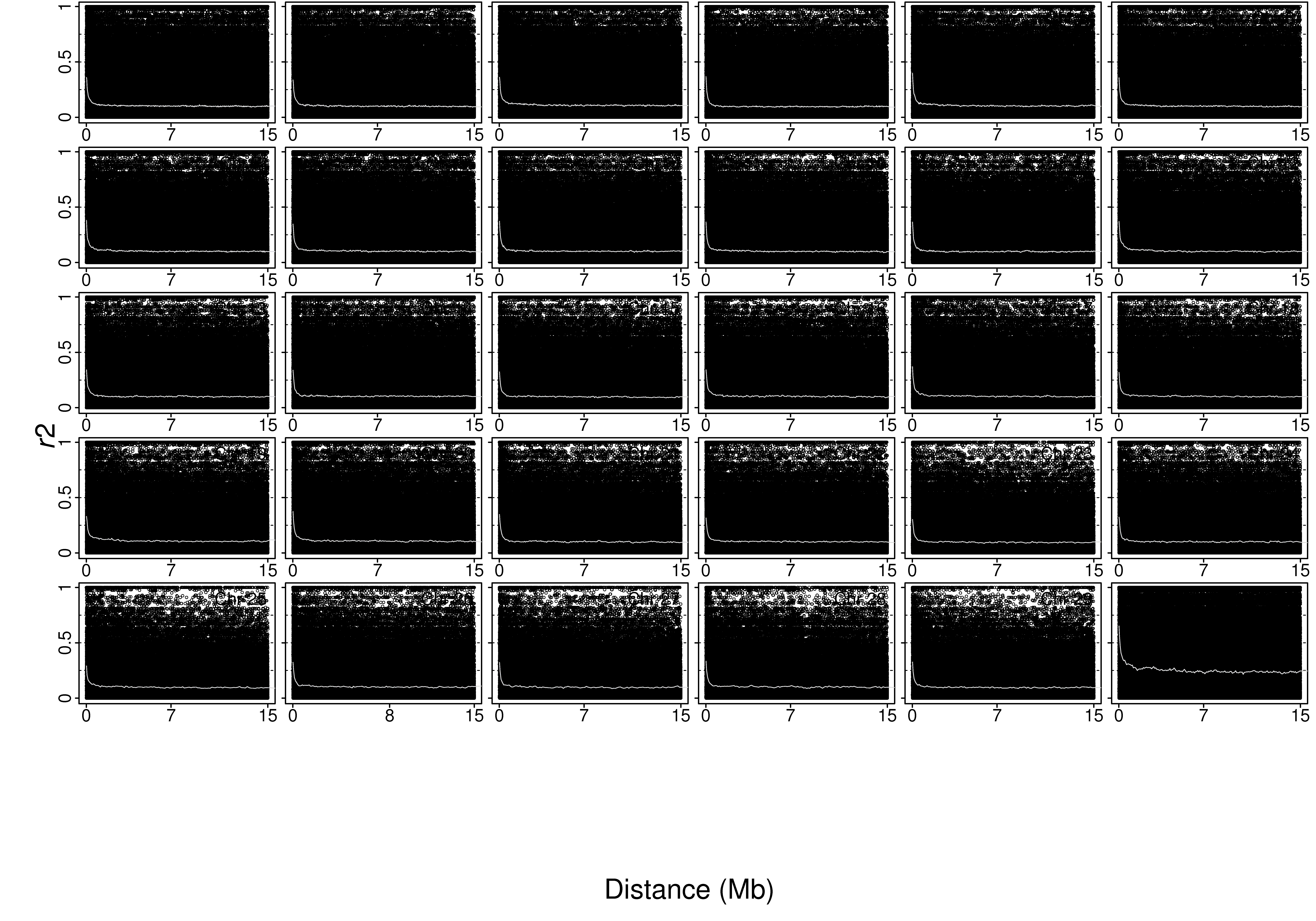

Supplement: Supplementary file 17 [file Image10.PNG]
