# Supplementary material for: Linkage Disequilibrium and Effective Population Size of Buffalo Populations of Iran, Turkey, Pakistan, and Egypt Using a Medium Density SNP Array
Source: Front Genet. 2021 Dec 7;12:608186. doi: 10.3389/fgene.2021.608186 (PMC8689148; doi:10.3389/fgene.2021.608186)
Supplement: Supplementary file 4 [file Table1.DOCX]

| TABLE 1: Wier and Cocerham | | | | | | |
| --- | --- | --- | --- | --- | --- | --- |
|  | NIL | ANA | EGY | AZI | KHU | MAZ |
| KUN | **0.024** | **0.039** | **0.053** | **0.049** | **0.057** | **0.077** |
| NIL |  | **0.038** | **0.052** | **0.047** | **0.055** | **0.073** |
| ANA |  |  | **0.037** | **0.011** | **0.021** | **0.042** |
| EGY |  |  |  | **0.046** | **0.054** | **0.073** |
| AZI |  |  |  |  | **0.020** | **0.039** |
| KHU |  |  |  |  |  | **0.045** |
